# Social groups and polarization of aesthetic values from symmetry and complexity

**DOI:** 10.1038/s41598-023-47835-w

**Published:** 2023-12-06

**Authors:** Kathleen B. Mather, Hassan Aleem, Yewon Rhee, Norberto M. Grzywacz

**Affiliations:** 1 Department of Psychology, Loyola University Chicago, Chicago, IL 60660-0804 USA; 2 Department of Molecular Pharmacology and Neuroscience, Loyola University Chicago, Chicago, IL 60660-0804 USA; 3 Department of Cognitive Science, Johns Hopkins University, Baltimore, MD 21218-2625 USA

**Keywords:** Neuroscience, Psychology

## Abstract

When deciding what images we prefer, our brain must weigh many aesthetic variables, such as symmetry and complexity. To date, aesthetic research has mainly focused on investigating one variable at a time. In this article, we use symmetry and complexity to study the problem of multi aesthetic-variable interactions. For symmetry and complexity, there are two simple interaction hypotheses. The independence hypothesis proposes that the evaluation of aesthetic variables is mutually independent. Meanwhile, Birkhoff’s aesthetic-measure hypothesis predicts that people prefer images high in symmetry and low in complexity, and dislike the opposite. To test these hypotheses, we generated images that systematically varied in levels of symmetry and complexity. We then compared the subjects’ preference maps to identify regions of likes and dislikes. Unlike the predictions from these hypotheses, we found that most, but not all subjects, formed two distinct natural clusters, termed “islands,” in terms of likes and dislikes. We also found that people with more art exposure were less likely to belong to an island. If someone did belong to an island, their gender influenced which cluster they belonged to. We discuss alternate hypotheses, possible mechanisms for the occurrence of islands, and their possible social implications.

## Introduction

Making a visual aesthetic judgment involves multivariate analysis across color, curvature, balance, symmetry, complexity, and many other properties. Some of these aesthetic variables have a direct relationship in images, while the associations between the others are less clear. For example, images higher in symmetry tend to exhibit more balance but less complexity^1–3^, whereas variables like color and curvature likely have less straightforward interaction. Thus, the multitude of visual aesthetic variables and their interactions present a challenge to understanding important aesthetic functions, like judgment, evaluation, and decision-making. How do we weigh different variables against each other when making an aesthetic judgment? Do they interact as independent variables, or do they exhibit complex nonlinear dependencies? The answers to these questions have important implications for understanding visual aesthetic preferences.

The interactions between various aesthetic variables and their influence on aesthetic preferences are relatively unexplored areas of research. Most existing research tends to focus on one primary variable, while holding the others constant^4, 5^. Although this approach is necessary in determining the individual contribution of each variable, it fails to account for the interactions between variables that contribute to ultimate aesthetic judgment. Therefore, one must design experiments that directly explore how multiple variables interact. One approach would be to use natural images^3, 6, 7^ because they contain multiple aesthetic variables. However, using real-world stimuli like figurative paintings or photographs has its own limitations. Such stimuli vary along many other unrelated factors such as semantic content, familiarity, and style, all of which could serve as confounding variables. An alternative is to control for such factors by creating an artificially generated set of images. This approach also allows for precise manipulation and examination of the desired variables.

Exploring symmetry and complexity may be a good starting point to study the interaction between visual aesthetic variables as they have been deemed key variables of aesthetic function^8, 9^. Aside from their individual impact that aesthetic research has focused on for decades, symmetry and complexity interact and compete in interesting ways, and thus, one should also study these variables together^10, 11^. For example, people differ in how they value symmetry and complexity and thus, weigh each variable differently to make an aesthetic judgment^1, 10–13^. This individuality begins with the tension between these variables^1–3^. The tension arises because the more symmetry an image has, the less information and thus, less complexity the stimulus yields. Hence, when an individual acquires aesthetic values, such tensions shape the learning process^14, 15^.

The interaction between symmetry and complexity is not well understood, though several hypotheses can be proposed. The simplest is the independence hypothesis, where symmetry and complexity interact as independent statistical variables. This hypothesis would predict that the most liked image would have a high level of symmetry^16–18^ and a moderate level of complexity^9, 19–21^. Alternatively, Birkhoff put forth an influential equation to explain how combinations of order and complexity elicit aesthetic preferences, calling this equation the aesthetic measure^16^. This equation predicts that people should like high levels of symmetry and low levels of complexity, and dislike the opposite. Research in some domains has provided some evidence for this equation^22, 23^. However, much of the emerging empirical evidence has suggested the interaction between symmetry and complexity is much more nuanced^22–24^. For example, a study that looked at symmetric and complex graphic patterns found that most participants prefer both symmetric and complex patterns, and not just one type^25^. In addition, the same study found that a portion of participants preferred non-symmetric and simple patterns, directly challenging Birkhoff’s views. A follow-up study using the same stimuli found that art experts tended to prefer asymmetrically complex patterns more than non-experts^26^. This result suggests an influence of experience and knowledge. Thus, while the existing research has provided interesting clues into the symmetry-complexity relationship, we need further systematic exploration that has well-designed stimuli and accounts for external factors. In particular, this further exploration should probe another yet untested prediction of Birkhoff’s theory. His equation predicts that the most disliked images are those with low symmetry and high complexity.

Previous research testing these hypotheses on the interaction between symmetry and complexity has been limited. Some research has examined this interaction with smartwatch screens^27^. While this work has high validity, it cannot fully explore the space of interaction because of the limitations in visual angle; a key bottleneck in the perception of symmetry^28, 29^. Other research has attempted to explore the entirety of this space by using artificially generated images that vary on both attributes^4^. While this research allows for more insights, its stimuli set splits symmetry into a limited binary variable. Another limitation of the existing studies is that they often do not incorporate the influence of personality and demographic variables. And investigators rarely check factors such as art exposure and expertise. Previous work shows that expertise modulates preferences for both symmetry and complexity^30^, but we need more investigation on art exposure. Additionally, personality^10, 31, 32^, and demographic variables such as gender^33, 34^ and age^33, 34^ have been shown to influence these preferences. Therefore, to understand aesthetic preferences fully, one must combine the inferences from a controlled set of stimuli, while accounting for external factors like art education, exposure, personality, and demographics.

In this study, we aimed to gain deeper insights into preference differences for images that varied systematically in symmetry and complexity. To do so, we generated images using an algorithm that produced unique and non-repeatable images with specific levels of symmetry and complexity. We gauged preferences across five sets of 49 images, representing combinations of 7 complexities and 7 symmetries. Repeating measurements allowed us to construct a detailed preference map for each participant. We then analyzed the statistical properties of this space to identify regions of likes and dislikes for each individual. Rather than taking a group-wise approach, we treated each individual separately. This allowed us to identify potential subgroups based on their likes and dislikes. This methodology enabled us to investigate whether there are natural groupings among humans based on their aesthetic preferences, even in a two-variable space. We also examined the impact of external factors such as art education, expertise, and exposure on the distribution of preferences. Finally, we studied the effects of personality and various demographic traits on this distribution.

## Results

### Individual responses

**Figure 1:**
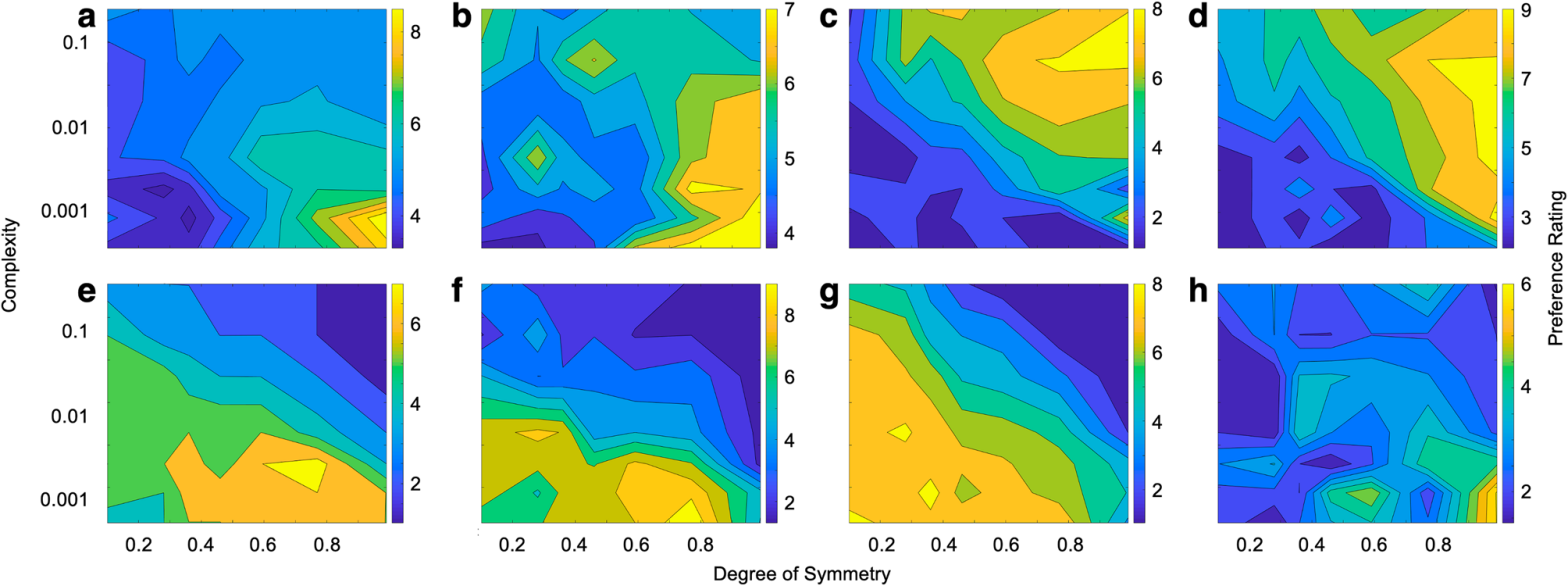
Contour plots of preference ratings of eight illustrative subjects (**a**–**f**) as a function of the degree of symmetry and complexity of the images. The likes and dislikes of the subjects are different, indicating high individuality. However, further inspection of individuals suggests that common patterns of preference may exist across individuals (see text for more detailed analysis).

Our main question was how different aesthetic variables interacted to influence aesthetic preferences. To answer this question, we developed visual stimuli with well-controlled symmetry and complexity statistics, and inspected the overall preference maps for each subject. Figure 1 shows examples of two-dimensional rating profiles for eight illustrative subjects.

The results showed individuality across subjects (Fig. 1). However, this individuality was such that preferences were not always as expected from our working hypotheses. For example, the independence hypothesis predicted that subjects’ most-liked statistics should normally consist of high degrees of symmetry^16–18^ and intermediate complexity^9, 19–21^. However, only Subjects c and d fulfilled this prediction. Alternatively, Birkhoff’s aesthetic measure hypothesis predicted that subjects’ most-disliked images should contain low degrees of symmetry. However, this prediction was not borne by Subjects e, f, and g. In turn, Birkhoff’s aesthetic-measure hypothesis^16^ predicted that subjects should like low complexity and high degrees of symmetry the most, and most dislike the opposite. Yet, the subjects shown in Fig. 1c–g challanged Birkhoff’s prediction for most-liked images, and every subject refuted his prediction for subject’s most-disliked images.

Another result unveiled itself in Fig. 1, as subjects a-d appear to exhibit a similarity in their most-disliked values of the aesthetic variables. Namely, these individuals mostly disliked images with low complexities and degrees of symmetry. These dislikes were in opposition to those of Subjects e–g, who did not care for high symmetry and high complexity. Hence, these seven subjects are suggestive of groupings of likes and dislikes. But if so, Subject h, with their much broader pattern of dislikes, demonstrates that these groups may not be applicable to every subject.

### Statistics of likes and dislikes

**Figure 2:**
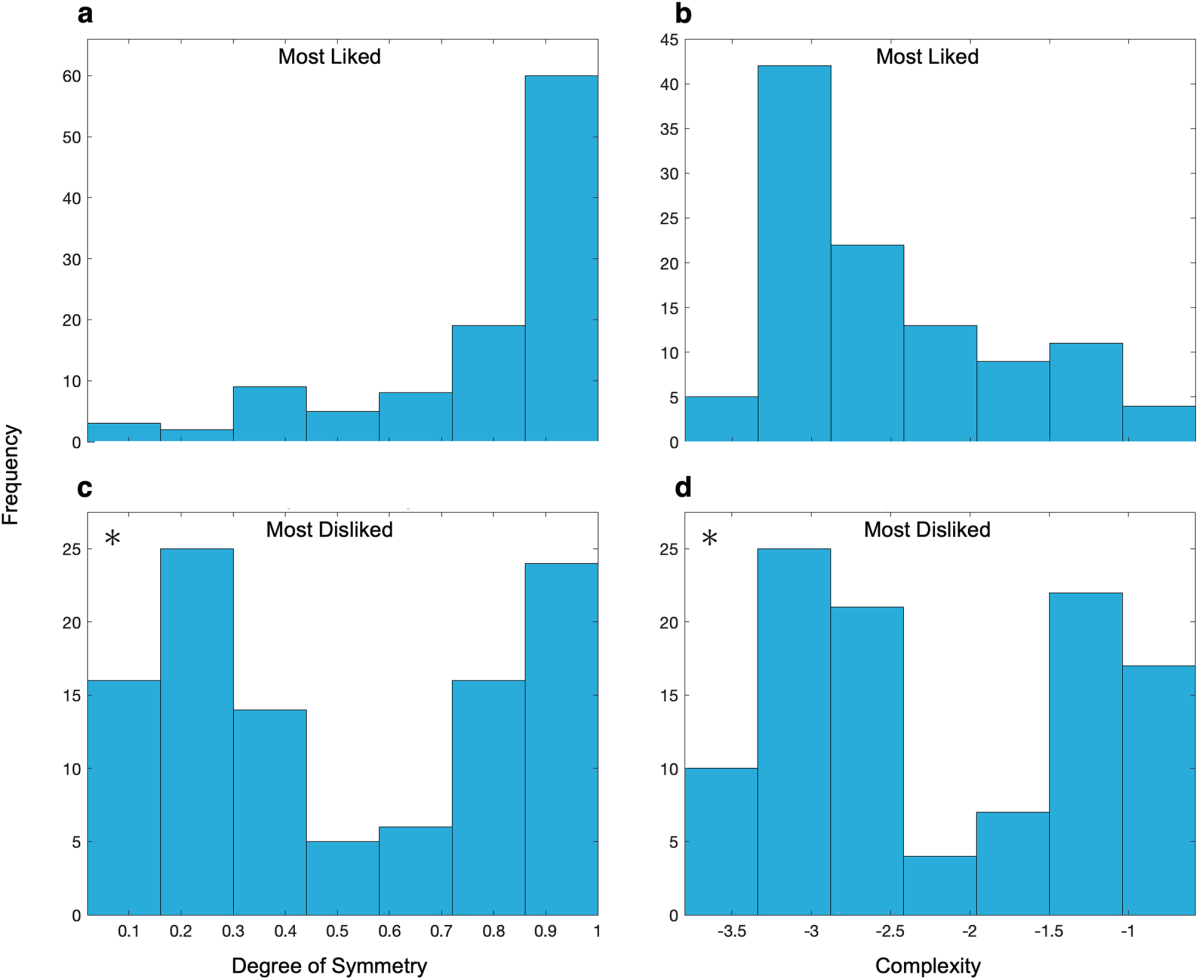
Histograms of most liked (**a**,**b**) and disliked (**c**,**d**) degrees of symmetry (**a**,**c**) and complexities (**b**,**d**). Although the “most-liked” histograms are unimodal, the “Most-Disliked” histograms are bimodal (asterisks representing statistically significant bimodality at the p < 0.001 level with the Hartigan’s Dip test).

Due to the potential groupings observed in Fig. 1, we designed further analysis to test the possibility of distinct clusters of likes and dislikes. To start, we measured the statistics of the most-liked and most-disliked pairs of degrees of symmetry and complexity. The simplest of these statistics were counts of these pairs over all the subjects. The estimate of each pair used robust statistics because the patterns in Fig. 1 did not resemble normal distributions. Histograms displaying the frequency of the most-liked and -disliked degrees of symmetry and complexity are shown in Fig. 2.

Figure 2a,b were exactly what we expected from the literature. The majority of people liked high degrees of symmetry the most^16–18^ and the preferred complexities exhibited a statistically significant rise-then-fall histogram, reminiscent of an inverted U-shape behavior^9, 19–21^. Thus, people disliked very low and high complexities, preferring intermediate ones. While our preference data is in accordance with the literature, the most-disliked data (Fig. 2c,d) are not according to the expectations from our working hypotheses. Figure 2c displays a bimodal distribution of dislikes for the degree of symmetry, suggesting that subjects exhibit a polarization between disliking low symmetry and high symmetry. This bimodality of dislikes is statistically significant (Hartigan’s Dip Test; Dip = 0.109; p < 0.001). The dislike of high symmetry by some people is surprising and, at first glance, contradictory to Fig. 2a. Similarly, Fig. 2d displays a statistically significant bimodal distribution of dislikes for complexities (Hartigan’s Dip Test; Dip = 0.116; p < 0.001). This bimodality suggests that subjects also show a polarization between disliking high complexities and low complexities. This bimodality similarly appears to violate the results of Fig. 2b.

Such bimodal distributions confirmed the violation of the expectations from our working hypotheses. The independence hypothesis predicted that Fig. 2c should have a single peak at low degrees of symmetry. In turn, Birkhoff’s aesthetic measure hypothesis predicted single peaks at low degrees of symmetry in 2c and high degrees of complexity in 2d. The significant bimodal distribution thus challenges both hypotheses.

The bimodality of symmetry dislikes (Fig. 2c) suggested another surprising conclusion. People seemed to divide themselves into two subgroups of preference profiles, confirming our initial observation from Fig. 1. People in one subgroup disliked low degrees of symmetry as expected^16–18^, but others disliked high symmetry. A similar conclusion was possible for the bimodality of complexity dislikes (Fig. 2d). We expected everyone to dislike low and high complexities because of the inverted U-shape behavior^9, 19–21^, but did know that some people disliked high complexities more than low ones and vice versa.

**Figure 3:**
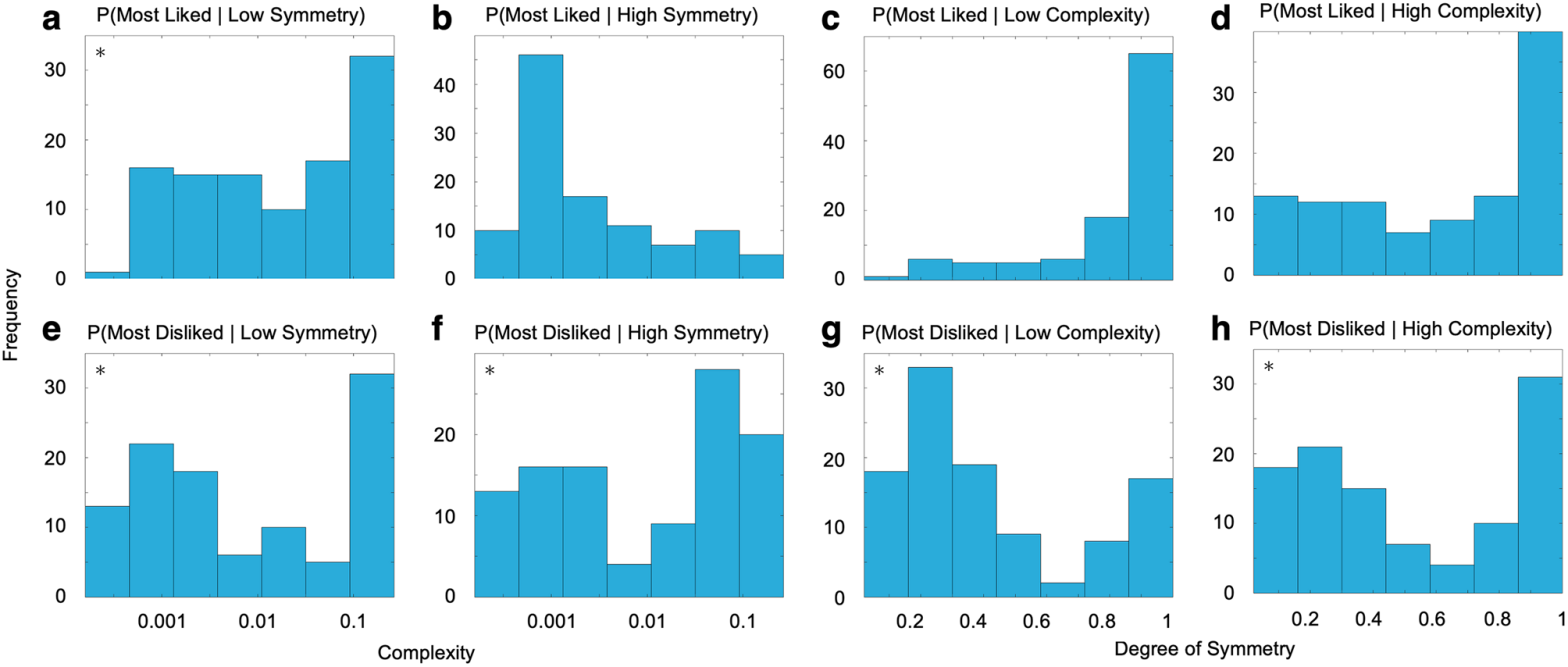
Histograms of most-liked (**a**–**d**) and disliked (**e**–**g**) degrees of complexities (**a**,**b**,**e**,**f**) and symmetry (**c**,**d**,**g**,**h**), contingent on low (**a**,**e**) and high (**b**,**f**) degrees of symmetry and low (**c**,**g**) and high (**d**,**h**) complexities. Although the majority of most-liked histograms are unimodal (**b**–**d**), all most-disliked ones are bimodal (asterisks representing statistical significance at the p < 0.001 level with the Hartigan’s Dip test).

When combining this conclusion with the analysis of Fig. 1, an even more intriguing picture emerged. Figure 1 revealed that the dependence of likes and dislikes in one variable might be contingent on the other variable. For example, Subjects a and b in Fig. 1 appeared to dislike low complexities only when the symmetry was low as well. To explore this possibility, we defined and measured second-order, that is, conditional statistics that would capture contingency in like-dislike behavior. The second-order statistics seen in Fig. 3 are conditional histograms that show subjects’ likes and dislikes given particular degrees of complexity and symmetry. As for Fig. 2, the calculations of these histograms have used robust statistics.

Five out of eight second-order distributions in Fig. 3 were significantly bimodal (Hartigan’s Dip Test; Dip > 0.116; p < 0.001). The histogram in Fig. 5a showed a bimodal distribution of most-liked complexities when presented with images with low degrees of symmetry. Therefore, a group of people preferred a higher complexity and another preferred a lower one under this condition. In contrast, unimodal distributions of most-liked complexities were seen when given a high degree of symmetry (Fig. 3b). Similarly, the distributions of most-liked degrees of symmetry were unimodal regardless of the complexity (Fig. 3c,d). On the other hand, all four histograms for the most-disliked parameters were bimodal regardless of contingency (Fig. 3e–h).

The contingency dependence in the histograms of Fig. 3 provided further evidence against the independence and Birkhoff-aesthetic-measure hypotheses. Neither could tackle the observed complex interactions of our experimental multivariate decision-making. In addition, the bimodality observations yielded another important, surprising implication. The distributions of the most-disliked image parameters appeared to exhibit bimodality more often than the distributions of most-liked parameters. This means that although aesthetic theories tend to emphasize what people like, the dislikes appear to be more discriminating, thus being important for aesthetic individuality.

### Islands of aesthetic values

The bimodality exhibited in the histograms of Figs. 2 and 3 suggested the possibility that subjects belonged to discrete aesthetic groups. How many such groups were present in our data? If we only had one bimodal histogram, we would have two groups: one of people in the lower mode and one in the higher. We had five bimodal histograms when considering the second-order data in Fig. 3. (We only used these second-order histograms because they were more detailed than those in Fig. 2, encompassing them.) Consequently, we could have anywhere from 2 to 32 groups when considering the combinations of people in each mode of the five histograms. Two groups would exist if people falling in a mode of one histogram (for example, the first mode of histogram 3a) would fall in identical modes in the others (for example, the second mode of 3e, the second mode of 3f, and so on). Thus, these people would show perfect correlation in terms of modes. In contrast, if no correlation existed, one would group people in 2^5^ = 32 separate ways. We determined the number of groups using the five-dimensional-vector technique described in Methods. As prescribed by the technique, we first ran a ${\chi }^{2}$ test to decide whether people had a random assignment to vectors, ruling out this null hypothesis (${\chi }_{31}^{2}=482.1;p<2\times {10}^{-16}$). We then ran post-hoc one-sided exact binomial tests to find the vectors with more people than expected. We found two pairs with two neighbor vectors each. By aligning the five components of the vectors with Histograms a, e, f, g, and h, the first pair was (1,2,2,1,1) and (1,2,1,1,1) ($p<3\times {10}^{-16}$ and $p<0.02$ respectively). In turn, the second pair was (2,1,1,2,1) and (2,1,1,2,2) ($p<6\times {10}^{-4}$ and $p<3\times {10}^{-7}$ respectively).

**Figure 4:**
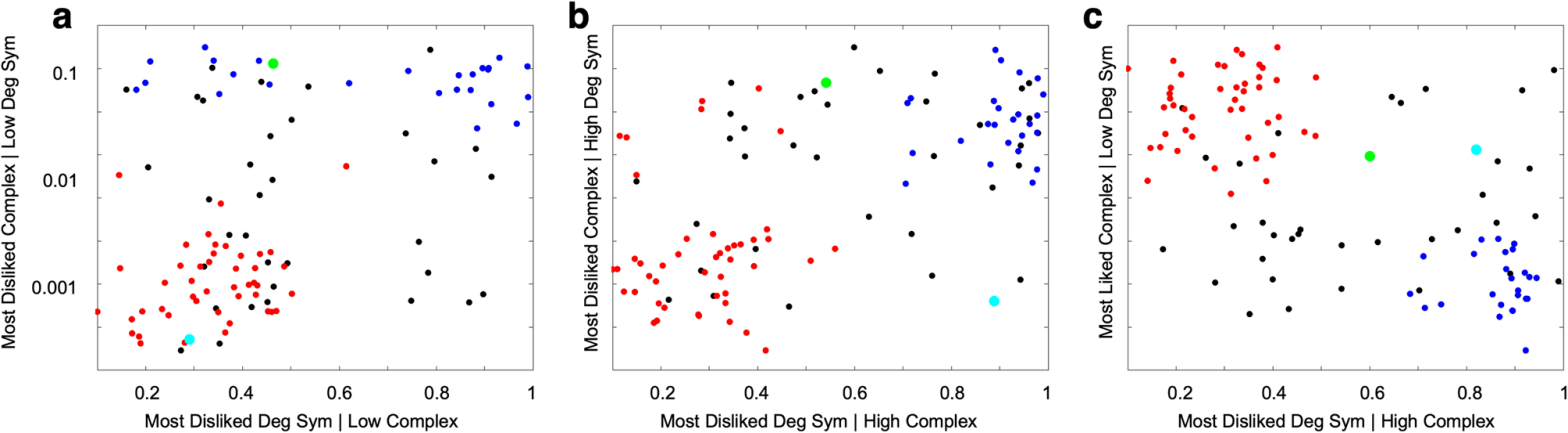
Three two-dimensional facets of the five-dimensional cube encompassing the two modes of the five binomial histograms in Fig. 3. Each dot represents a subject in our experiment. The red and blue dots are people in the two significant discrete groups in the data. Because these groups are in isolated regions of the five-dimensional cube, we call them islands. The black dots are people who we cannot place in islands in a statistically significant manner. The cyan and green dots are examples of people not in islands to illustrate how they did not contain all the individuals. The green dot appears to be in the blue island in (**a**) but not in (**b**,**c**). The cyan dot appears to be in the red island in (**a**) but not in (**b**,**c**).

Figure 4 plots the data for all the people in these two discrete aesthetic groups and outside them. Because the vectors are the 32 corners of a five-dimensional cube, displaying them directly is not possible. Instead, we use the relatively recently developed technique of multifaceted visualization^35, 36^. To explain this technique, imagine that the location of a person has five coordinates, say (x_1_,x_2_,x_3_,x_4_,x_5_). One can fully represent this location by projecting it to three facets of the cube, say (x_1_,x_2_), (x_3_,x_4_), and (x_1_,x_5_). Thus, these three graphs hold all the information on the location.

The subjects could be divided in a statistically significant manner into three groups, colored in red, blue, and black in Fig. 4. The red and blue dots represented people in the two significant discrete groups in the data. Because these people were in non-overlapping regions of the five-dimensional cube, we referred to these groups as islands. The black dots were people who we could not place in islands in a statistically significant manner. We tested reliability by checking whether people in each island stayed in it over time. For this purpose, we measured the joint probability of a person being in the same quadrant of Fig. 4a–c in Trials 1 and 5. We found this probability to be $0.78\pm 0.06$ in the red island. For the blue island, the equivalent probability was $0.92\pm 0.05$. These probabilities were significantly higher than the results obtained by placing people at random quadrants in Fig. 4a–c. This suggested that island membership was stable within the testing period. That some people in the islands appeared to be outside them in some trials was not surprising given the noise in the data and the instability of aesthetic values^34^. Relatedly, the preferences of people outside the islands were even more unstable. The joint probability to be outside the islands in the first and fifth trials was only $0.25\pm 0.07$.

Most subjects (66%) divided themselves between the two islands (Binomial Exact Test, n = 106, p < 0.0004). Of those subjects that were in the islands, the majority (64%) was in the red-labeled island (Binomial Exact Test, n = 70, p < 0.006). Some subjects not belonging to islands could appear in a facet near individuals belonging to islands. Two examples were the subjects marked in cyan and green in Fig. 4a. However, the same subjects appeared outside the islands when probing other facets as shown in Fig. 4b,c. The same did not happen to members of an island because they were always near each other regardless of the facet.

**Figure 5:**
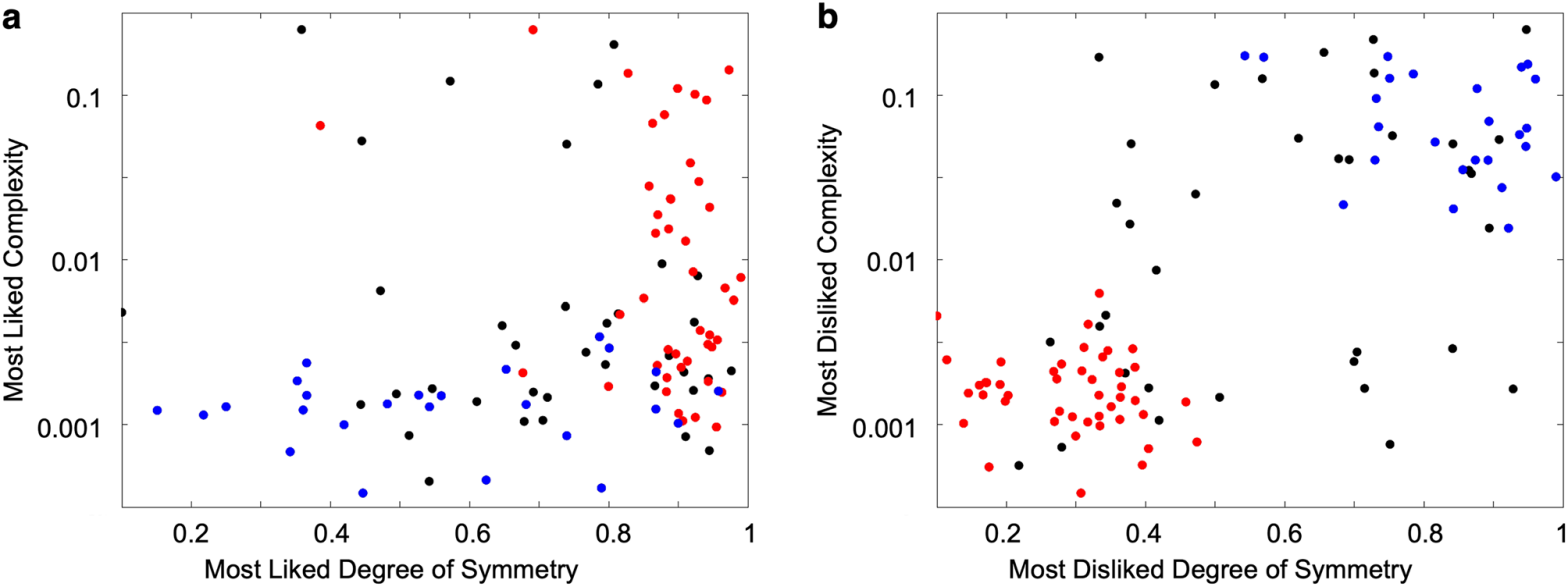
Most-liked (**a**) and most-disliked (**b**) Symmetries and complexities for all subjects categorized as in Fig. 4. Red-island subjects most-like high degrees of symmetry, and most-dislike the combinations of low degrees of symmetries and complexities. Blue-island subjects most-like low complexities, and most-dislike the combinations of high degrees of symmetries and complexities.

Having established the existence of islands, we then explored their values associated with subjects in each group. To do so, we plotted in Fig. 5a the most-liked complexities and degrees of symmetry for subjects in the red and blue islands. Similarly, we plotted in Fig. 5b the most-disliked complexities and degrees of symmetry. The people outside the islands also appeared in these figures.

Members of the red island tended to like high degrees of symmetry the most (Fig. 5a). Specifically, these people preferred all ranges of complexity if the degree of symmetry was high and thus, we called this island the Symmetry-Island. These people also most-disliked the combinations of low complexities and degrees of symmetry (Fig. 5b). In turn, members of blue island tended to most-like low complexities (Fig. 5a). Specifically, these people preferred all ranges of degree of symmetry if the complexity was low and thus, we called this island the Simplicity-Island. People in this island most-disliked the combinations of high complexities and degrees of symmetry (Fig. 5b).

Interestingly, the preferences for both islands overlap to some degree, while the dislikes exhibit polarization. Most preferences lie in the bottom right corner in Fig. 5a. This is consistent with Birkhoff’s aesthetic measure hypothesis, which predicts that people should like high levels of symmetry and moderately high levels of complexity. However, the dislikes are polarized (Fig. 5b) and not clustered in the upper left, which are opposed to the predictions using Birkhoff’s equation. Instead, Fig. 5b shows that those in islands tend to dislike combinations of either low complexity and symmetry or high complexity and symmetry. Therefore, people’s dislikes tend to represent a positive correlation between complexity and degree of symmetry.

**Figure 6:**
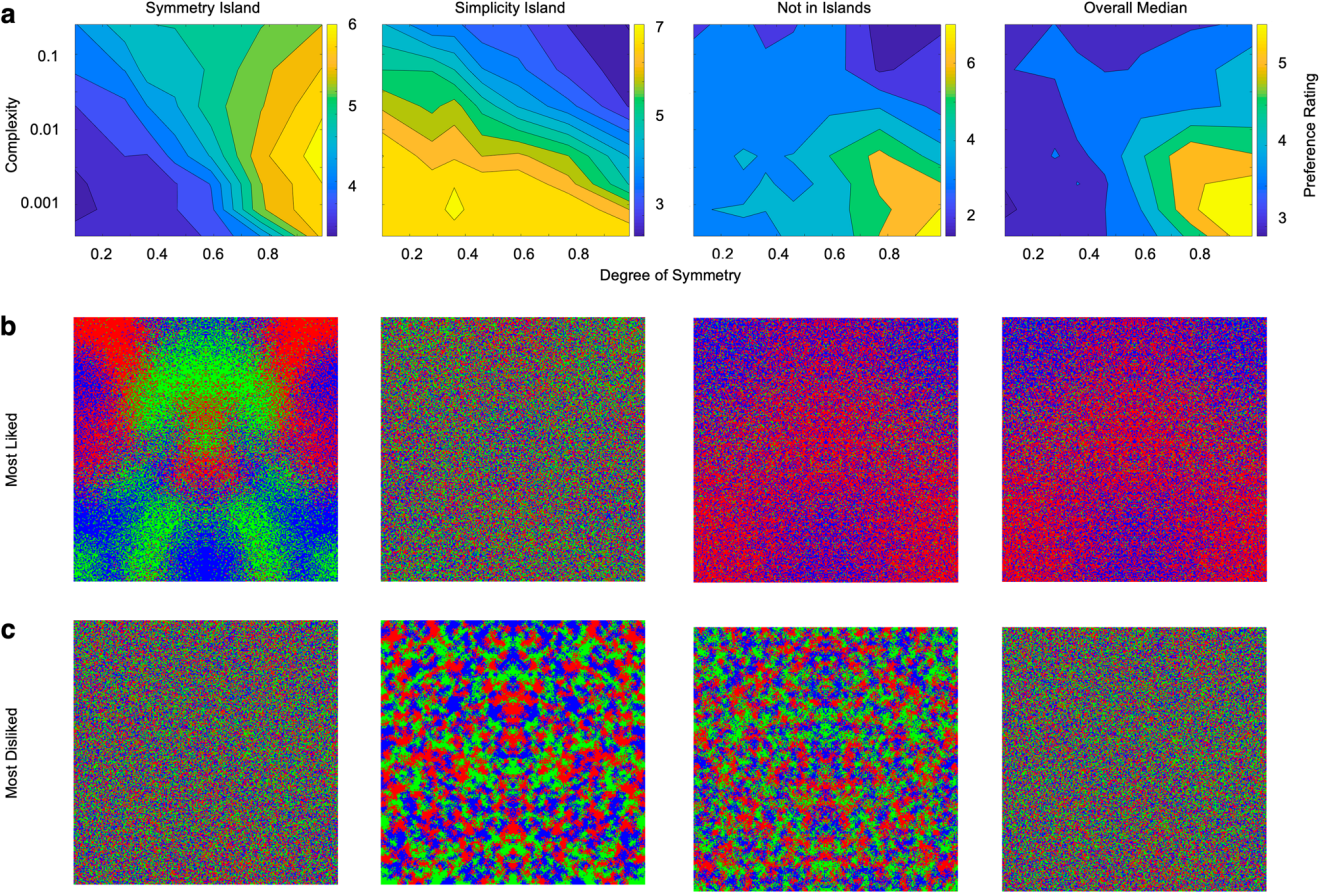
Median contour plots (**a**) and examples of most-liked (**b**) and most-disliked (**c**) images for symmetries and complexities for all subjects, subjects in the symmetry-island, subjects in the simplicity-island, and subjects not in islands.

To understand these preference behaviors better, we looked at the median contour plots for people in the Symmetry- and Simplicity-Islands, for those not in islands, and for all people together (Fig. 6). The contour plot for the Symmetry-Island shows a liking for high degree of symmetry regardless of complexity (Fig. 6a). This preference for high symmetry is evident in the typical image that this group most-likes (Fig. 6b). Moreover, this group dislikes the combination of low complexity and degree of symmetry (Fig. 6a,c). In contrast, people in the Simplicity-Island show a median liking for low complexity regardless of the degree of symmetry (Fig. 6a,b), while disliking the combination of high complexity and degree of symmetry (Fig. 6a,c). Comparing the Symmetry- and Simplicity-Islands, the polarization of dislikes is clear. Interestingly, people not belonging to islands have a narrow region of median liking for symmetry and simplicity (Fig. 6a,b), while their median dislikes are spread broadly, with a slight emphasis on high complexity and degree of symmetry (Fig. 6a,b). Finally, we observe a similar preference in the entire sample as those not in islands (Fig. 6a,b). However, the dislikes are more focused on either high complexities or low degrees of symmetry (Fig. 6a,c). Overall, although people’s dislikes are different, they tend to prefer images with low complexity and high symmetry.

When the complexity was low for a given image, it created images dominant in one or two colors (two right panels of Fig. 6b). Because preference to color was highly individual^34^, we wanted to know if it had a significant effect on the islands. To do so, we looked at color preference and found that most images had color distributions that were statistically indistinguishable from a random one. The only exceptions were images with low complexity and high degree of symmetry (two right panels of Fig. 6b). As seen in Fig. 5b, these images did not contribute to the islands. Furthermore, even with different predominant colors and across subjects, these images never were among the most disliked.

### Determinants of membership in islands

**Figure 7:**
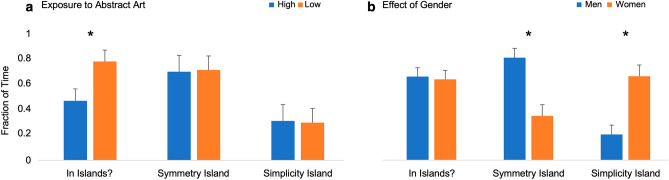
Fraction of time (probability) that people from two categories are in islands as opposed to not in them, and in the symmetry-island as opposed to in the simplicity-island. (**a**) Results parametric on the amount of exposure to abstract art. The categories of people are high exposure (blue) and low exposure (orange). (**b**) Results parametric on gender. The categories of people are men (blue) and women (orange). Asterisks represent statistically significant differences. The data show that people with low exposure to abstract art tend to be in the islands significantly more often than do people with high exposure. Furthermore, men to be in the symmetry-island more than women and vice versa for the simplicity-island.

The visual-stimuli ratings discovered and provided insight into aesthetic islands of preference found with our subjects. These findings prompted further investigation of what was moderating these islands. In particular, we studied what factors could be contributing to the occurrence of islands by examining demographic, personality, education, and art-exposure information. Despite the large number of surveys that we used to explore these variables, only two showed relevance to the islands. Figure 7 illustrates these factors and their effects.

We found that art-exposure had a significant effect on belonging to an island (Fig. 7a). Subjects with low art exposure tended to be in an island more often than subjects with high exposure. In particular, abstract art exposure had a statistically significant effect on placing people in islands (Exact Binomial Test, p < 0.003). Low exposure to figural art also appeared to have an effect, but the statistics were borderline. Interestingly, while art exposure impacted whether someone belonged to an island, art education and expertise did not. We also observed no significant impacts of personality, demographics, gender (Fig. 7b), education, and social-media exposure and use in belonging to an island. Importantly, exposure to art also did not impact which island a person was in.

Given that some people belonged to islands, we further examined what moderated the systematically differing preferences between the Symmetry- and Simplicity-Islands. We observed a significant effect of gender on what island subjects belonged to, as seen in Fig. 7b (${\chi }_{1}^{2}=10.7;p<0.002$, with Yates’ Continuity Correction). Men tended to be in the Symmetry-Island more often, while women were more prominent in the Simplicity-Island. Therefore, the biggest factor in our study determining which island of preference one belonged to was gender. We saw no significant effects of personality, demographics, education, art-exposure, art expertise, and social media exposure and use.

## Discussion

How do aesthetic variables interact when one performs aesthetic functions? In this study, we tested two working hypotheses by comparing preferences for images with varying degrees of symmetry and complexity. The first hypothesis suggested that the evaluation of aesthetic variables was mutually independent, meaning the most liked combination would have the optimal symmetry and complexity, and vice versa. Further, we predicted that the combination of the participants’ most-disliked degrees of symmetry and most-disliked complexities yielded the least preferred stimuli. A second hypothesis came from Birkhoff^16, 23, 24^, who proposed that aesthetic preference for an image arose from the ratio between its order and complexity. Therefore, he predicted that high symmetry and low complexity images would be the most liked. His equations also predicted that low symmetry and high complexity images would be the most disliked. Neither the independence nor Birkhoff’s hypotheses held up to our experimental scrutiny. Instead, our experiments revealed that the likes and dislikes of participants fell into two unique clusters in the multivariable space; we termed them “islands.” Further evidence for clustering into sub-groups of aesthetic values has been discussed elsewhere the context of complexity^21, 37^. We think of these islands as “social” groups for three reasons: first, the definition of the word “social” is, “relating to society or its organization”^38^. The islands that we have uncovered are a temporally consistent organization of likes and dislikes in a large group of individuals. Second, we show that a major part of this organization is the difference between the genders, another key social factor. Third, members of society with exposure to arts appear to be immune from being in the islands.

Although the first proposed aesthetic measure is that of Birkhoff, several other theories have appeared in the literature^23^. The organization of aesthetic preferences in polarized social groups is so surprising that it also rules out all these later aesthetic measures. Another surprise is finding that some people dislike high degree of symmetry under certain conditions. This is surprising given the significant historical emphasis on symmetry for beauty^4, 39^. Others have reported on the non-universality of the law of symmetry^26^ in line with our findings. Our unanticipated results do not invalidate past findings (Fig. 2a,b). Rather, we build on them by allowing a more intricate look into aesthetic ratings by considering what people dislike. Analyzing subjects’ dislikes aided to finding islands because of their polarizization in dislikes (Fig. 2c,d). Furthermore, our results suggest that analyzing the interaction of multiple aesthetic variables can reveal intricacies unique to more realistic conditions.

Why do aesthetic-variable interactions give rise to polarized social groups? We cannot yet offer an answer, but some of our results and those of others suggest possible explanations. Of the myriad of factors that we explored, only one correlated with membership to aesthetic islands: art exposure. People with high art exposure tended to be outside of islands. This finding suggests that being in an island is the natural state for individuals without high art exposure. Perhaps, broad art exposure may inject variability to aesthetic-value learning, nudging people away from the islands and providing opportunities to enrich the range of preferences. Another reason for people being outside the islands may be that societies also have anti-conformists^40, 41^. These individuals may also be outside the islands, expanding the category beyond those with high art exposure. This expansion is important because art exposure only offers a partial explanation for island membership/formation. For example, 23% of people with below average art exposure are outside the islands in our data. Similarly, 46% of people with above average art exposure are in the islands.

The occurrence of polarized social groups can also be understood more generally in one of two ways: first, people have a natural tendency to form groups/islands by conforming to perceived social norms^42, 43^. Such a tendency has biological roots^40, 44^, evolutionarily stemming from the social support that people receive by peers in the group^45, 46^. Consequently, people may be more influenced by common aesthetic themes observed in their social circles. Second, the emergence of islands can potentially be attributed to a combination of biological and cultural factors. Our findings revealed a notable gender division within the islands, with men predominantly belonging to the Symmetry-Island and women to the Simplicity-Island. These gender differences stood in our experiments as the sole significant effect associated with island-specific membership. Interestingly, personality traits did not show significant correlations, contrary to prior literature suggesting the influence of openness on art preference^47^. This discrepancy may be due to the use of abstract stimuli in our study.

Importantly, distinguishing between sex and gender^48^ as contributors to islands has proven challenging because the discrepancy between the two is less than 5%^49^. Future research could address this limitation by controlling for sex and gender differences. Such control could provide insights into whether cultural factors play a more substantial role than biological factors in shaping the emergence of these aesthetic islands. We see three possible ways by which biological and/or cultural factors may affect gender influences on island membership: First, women have been shown to be more risk averse than men^50, 51^. Part of women’s increased risk-aversion is biological^52^ and part is cultural^50, 53^. Both parts may stem from the added social vulnerability of women throughout history^50, 53^. Examples of reasons for the added vulnerability include the risk of domestic violence, having to raise children alone, lack of control over familial resources, and lower pay for equal jobs. Interestingly, a recent study has found a positive correlation between risk-aversion and the aesthetic preference for low complexity^14^. Second, men have been shown to value physical beauty more than women^54^. In contrast, women place higher value in other factors of mate selection such as personality traits^54^. Therefore, given the positive correlation between symmetry and facial attractiveness^55, 56^, men may have a stronger bias toward symmetry than women do. Third, other explanations involve evolutionary or developmental differences between men and women based on their distinct social roles. For example, a study suggests that the different neural aesthetic circuitries used by men and women reflect an evolutionary distinction stemming from the division of labor between them in hunter-gatherer times^57^. A related explanation reflects differences in developmental environments such as social norms in toys^58, 59^, parenting styles^60, 61^, and gender expectations^62, 63^. However, although these latter explanations are of interest, we have found no clarifying power in them with respect to our results on symmetry and complexity.

The aesthetic-island observation suggests profound implications for how we understand individual and social dynamics. Although our focus is on aesthetic values, the process of learning and development leading to polarized social groups may apply to any value system. Herd mentality, that is, how values are influenced by a larger group, is often considered an inevitable consequence of human social behavior^64^. Our results suggest that this phenomenon mostly stems from what people dislike and not what they like, leading to polarization. However, our results also show that greater exposure to art is correlated with more individualistic likes and dislikes outside the islands. Therefore, these findings suggest that the increased exposure enriches the building blocks with which individuals construct their models of aesthetic appreciation. Perhaps, such increased exposure also works outside aesthetics. Exposure to diverse ideas may be an individualization factor, promoting self-determined values.

## Methods

### Participants

One-hundred-and-ten participants took part in the experiment, all giving informed consent, receiving $9 per hour for participating, and taking 39.5 min, on average, to complete the test. We discarded four of these participants after failing to meet attention checks, which consisted of entering the exact same preference rating for five or more consecutive images. The remaining 106 participants consisted of 53 women and 53 men, had ages ranging from 20 to 61 years old (mean = $$26\pm 8$$), and were from countries mostly in Latin America, Europe, and Africa. We used Prolific (https://www.prolific.co/), an online participant recruitment platform, to recruit subjects. This experiment was approved by the Loyola University Institutional Review Board. All experiments were performed in accordance with relevant named guidelines and regulations.

### Testing conditions and stimuli

The experiment was conducted entirely online. It was created inPsychoPy3 version 2023.1.0^65^ and then hosted on Pavlovia.org (https://pavlovia.org/) to be distributed to participants through Prolific. Before the experiments, each participant filled out a consent form along with demographic information with Qualtrics (https://www.qualtrics.com/). A battery of surveys was administered on the same platform as the last session.

All participants used their own devices to complete the experiment; only desktop computers were permitted. The stimuli were presented in a way to maintain relative positions and proportions to the size of each participant's screen.

The visual stimuli used in the experiments were created using MATLAB (The MathWorks Inc., Natick, Massachusetts). Each image consisted of a $300\times 300$ pixels array. Each image varied across two parameters, namely, the degree of symmetry and complexity, both being numbers from 0 to 1 defined elsewhere^1^. The algorithm that generated the stimuli first seeded three pixels, one red, one green, and one blue, in random positions of the image. Afterwards, the algorithm continued to paint the remaining pixels one by one until all of them were red, green, or blue. The process of painting after the initial seeding was as follows:

1. Randomly select a pixel from among those not yet filled;
2. Count ${n}_{r}, {n}_{g}, {n}_{b}$, namely, the numbers of red, green, and blue pixels within a radius of our selected pixel; we call this radius “the radius of integration”:
3. If ${n}_{r}+ {n}_{g}+ {n}_{b}=0$, then select the color of this pixel at random from among red, green, and blue;
4. Otherwise, calculate the probabilities $\frac{{{p}_{x}=n}_{x}}{\left({n}_{r}+ {n}_{g}+ {n}_{b}\right)}$, where $x\in \left\{r,g,b\right\}$;
5. Choose the color of selected pixel at random according to the probabilities ${p}_{r},{p}_{g},{p}_{b}$;
6. Consider the pixel where our selected pixel would be if the image was reflected about the central vertical axes of the image;
7. Fill the symmetrically positioned pixel with the same color at our original selected pixel with probability equal to the degree of symmetry (defined elsewhere^1^);
8. Otherwise, choose the color of symmetrically positioned pixel at random according to the probabilities ${p}_{r},{p}_{g},{p}_{b}$;
9. Go back to Step 1.

The radius of integration in Step 2 is connected to the notion of complexity, which is commonly used in studies of visual aesthetics^5, 9, 10^. Whether one defines complexity as the number of objects in an image^66^ or in terms of information theory^9, 67^, the complexity of our visual stimuli increases when the radius of integration decreases. Calculations show that the complexity has an asymptotic behavior that decreases with the square of the radius of integration (Supplementary Information: https://osf.io/adbkt). Hence, the graphs in this article use the following approximation for complexity: $C=1/{r}^{2}$, where $C$ is the complexity and $r\ge 1$ is the radius of integration in pixels.

**Figure 8:**
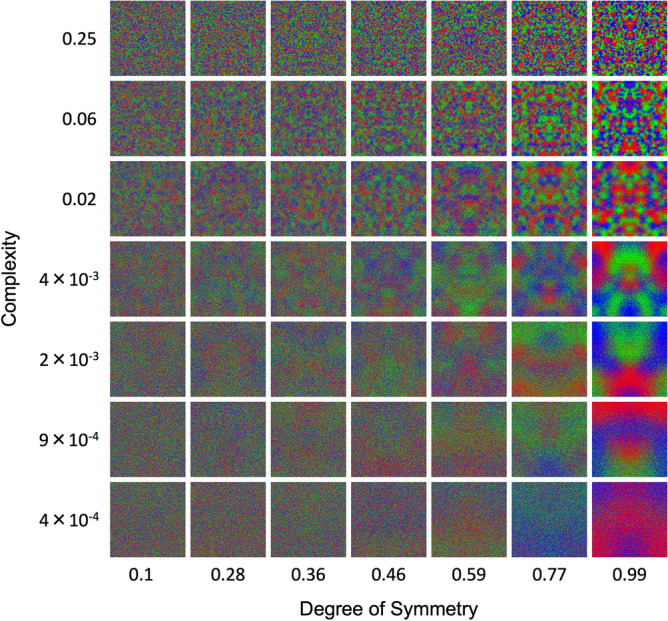
Examples of the computer-generated stimuli used in our experiments. The degree of symmetry is represented on the horizontal axis. In turn, we represent the complexity on the vertical axis.

We used a 7 × 7 stimulus space, comprising 7 complexities and 7 degrees of symmetry. The complexities were $0.25, 0.063, 0.020, 4.4\times {10}^{-3}, 1.9\times {10}^{-3}, 8.7\times {10}^{-4},$ and $3.9\times {10}^{-4}$. In turn, the degrees of symmetry were 0.1, 0.28, 0.36, 0.46, 0.59, 0.77, and 0.99. This led to the production of 49 images in one batch (Fig. 8). A total of five independent batches of 49 images were created, each having identical parameters, however being distinct due to the generative nature of the algorithm. During the experiment, a unique batch was shown per session, allowing us to take repeated measurements of underlying statistical parameters without ever truly repeating an image.

All the visual stimuli used in our experiments can be found alongside the experiment code and raw data at https://gitlab.pavlovia.org/haleemer/ct6.

### Procedure

After giving their consent, participants received a Pavlovia link to participate in the experiment. The experiment procedure was as follows: first, participants were shown a small unique sample of the type of images they would later be rating. The purpose of this phase was to acclimate the subjects to the visual stimulus to calibrate their ratings to the space of stimuli. Next, participants moved on to the rating sessions. There was a total of five rating sessions, each with a unique batch of images randomized across participants. In each session, participants saw images from one batch of 49 images in a randomized order. In each trial, we asked participants to rate the image by clicking on a continuous Likert scale from 1 to 10, with 1 meaning they strongly disliked and 10 meaning they strongly liked the image. After placing their rating, participants were asked to click a ‘submit’ button on the top of the screen away from the scale, this completed one trial. The purpose of the click to advance ‘submit’ button was to prevent anchoring bias, prompting users to approach the rating scale from a neutral physical location on each trial.

After completing the rating portion of the experiment, participants were directed to Qualtrics, where they completed the Big Five questionnaire^68^, the International Personality Item Pool^69^, as well as a series of questions that probed demographics, education, and art interest and exposure (Supplementary Information: https://osf.io/adbkt). The demographics included age, gender, country of origin, and native language among other factors. In turn, the educational part of the survey asked about one’s highest degree and art-related studies. Finally, the art-interest survey probed exposure to different forms of art.

### Data analysis

We began by calculating the median dependence of each subject’s preference ratings. This median was a function of the two-dimensional variable comprising complexity and degree of symmetry. We then calculated the values of this two-dimensional variable that yielded the highest and lowest ratings for each subject. This was done by determining the median complexities and degrees of symmetry of each subject’s five top- and bottom-rated stimuli ($\approx$ top and bottom deciles). We termed these values “first-order statistics.” This was because they referred to the most-liked and most-disliked complexities and degrees of symmetry in the entirety of each subject’s dataset. Although we used a categorical technique, complexities and degrees of symmetry were continuous variables. The purpose of performing such categorization was to obtain a robust statistical estimation of the most-liked and most-disliked positions.

Next, we calculated “second-order” statistics. They involved similar robust calculations as for the first-order statistics but adapted to consider contingencies on the lowest or highest values of complexities and degrees of symmetry. The definition of these values was the 3 highest or lowest complexities or degrees of symmetry out of a possible 7. With a fixed contingency, we had 21 ratings (for example, the 3 highest complexities times 7 degrees of symmetry). We then estimated the highest or lowest rating by using the top or bottom 3 out of 21 ($\approx$ top or bottom 15^th^ percentile). Given the four contingencies (high/low complexity, high/low symmetry) and two possible outcomes (most-liked, most-disliked), we obtained eight second-order datasets. These sets were besides four first-order datasets (combinations of complexity and degree of symmetry with most-liked and most disliked).

Finally, we examined the distributions of the first- and second-order statistics. The occurrence of multimodal distributions would provide evidence for separate subgroups of preference among subjects. However, unimodal distributions would suggest that the population consists of a single group. To test the null hypothesis of unimodality, the Hartigan’s Dip test^70^ was used with a threshold probability of 0.0xx to account for multiple tests.

If participants had multimodal distributions, this would suggest that they belonged to non-overlapping groups of preference. To test this hypothesis, each participant was assigned an N-component binary vector. It indicated the mode the participant belonged to in each of the N second-order sets exhibiting bimodality. Hence, participants divided themselves into the 2^N^ corners of a N-dimensional cube. To inquire whether the data showed participants belonging to non-overlapping groups, we first used a χ^2^ test. We apply it to the null hypothesis that all our participants were equally likely to fall in any of the 2^N^ corners. We then tested the null hypothesis for each corner of the cube that the probability that any individual would fall there was equal or smaller than 1/2^N^. This probe involved the Exact Binomial test. If the probe yielded a probability that was much higher than expected, we would have evidence of non-overlapping groups of preference. Because subjects would thus be in non-overlapping regions of the N-dimensional cube, we refer to these putative groups as islands.

We then investigated whether any of the personality, demographics, education, or art interest/exposure factors could explain island membership. For any of these factors, we first probed inhomogeneities in the distributions with χ^2^ tests. If we detected an inhomogeneity, we then used Exact Binomial tests to test the direction of the discrepancy. In all these tests, we made sure to use stricter significance thresholds (p < 0.001) for individual comparisons, to compensate for the number of inferences.

## Data Availability

The data that support the findings of this study are openly available on the Open Science Framework (OSF) at https://osf.io/adbkt.
